# Integration of ^68^Ga-PSMA-PET imaging in planning of primary definitive radiotherapy in prostate cancer: a retrospective study

**DOI:** 10.1186/s13014-016-0646-2

**Published:** 2016-05-26

**Authors:** Sabrina Dewes, Kilian Schiller, Katharina Sauter, Matthias Eiber, Tobias Maurer, Markus Schwaiger, Jürgen E. Gschwend, Stephanie E. Combs, Gregor Habl

**Affiliations:** Technische Universität München (TUM), Klinik und Poliklinik für RadioOnkologie und Strahlentherapie, Ismaninger Straße 22, 81675 Munchen, Germany

**Keywords:** PSMA-PET, Prostate cancer, Radiation therapy, Radiation oncology, Treatment planning

## Abstract

**Background:**

Prostate cancer (PC) is one of the most commonly treated cancer entities with radiation therapy (RT). Risk group-adapted treatment and avoidance of unnecessary toxicities relies primarily on accurate tumor staging. Thus, the introduction of prostate-specific membrane antigen (PSMA) in diagnosis and treatment of PC is a highly interesting development in radiation oncology of urologic tumors. The present work is to evaluate the integration of ^68^Ga-PSMA-PET imaging into standard radiation planning of primary definitive treatment of PC and to determine the impact of PSMA imaging on tumor staging.

**Methods:**

The data of 15 patients treated for PC between August 2013 and April 2015 were evaluated. Treatment planning included ^68^Ga-PSMA-PET imaging. We analyzed whether the use of PSMA-imaging led to a change of the TNM stage and if it influenced the RT treatment approach or the target volume, due to changes in the gross tumor volume (GTV) or clinical target volume (CTV), in the final treatment plan.

**Results:**

In 53.3 % of the analyzed patients a change occurred in the TNM stage based on ^68^Ga-PSMA-PET examination. The RT concept changed in 33.3 % of all patients, leading to relevant changes in the planning target volume. Among these, an additional irradiation of the pelvic lymph drainage due to tracer uptake in lymph nodes was performed in 25 %. Furthermore, boost volumes of PET-positive lymph nodes were added in 80 % of these cases. A down staging due to the ^68^Ga-PSMA-PET examination occurred in 13.3 % of all cases.

**Conclusions:**

The integration of ^68^Ga-PSMA-PET-imaging into the RT treatment planning process can be useful for detailed target volume planning. The performance of a ^68^Ga-PSMA-PET frequently leads to changes in the TNM stage, altering the RT treatment regimen and the target volume. A prospective trial is underway to evaluate the impact of ^68^Ga-PSMA-PET based treatment planning on outcome.

## Background

Prostate cancer (PC) is the most common tumor entity in males in many developed countries [1]. Almost 65.000 patients were newly diagnosed with PC in Germany alone in 2011 and every year over 10.000 die from it [2]. There are several treatment options for localized PC, among them radiation therapy (RT), which may be administered alone or in combination with hormonal therapy [3]. Definitive RT for PC relies primarily on accurate clinical and radiological tumor staging. To differentiate between local, regional, or systemic disease, initial staging provides the basis for further treatment decisions and enables risk group-adapted treatment. On the one hand staging is of paramount importance to increase the curative chance of patients and on the other to spare them from unnecessary toxicities. Several imaging modalities are used for staging PC such as CT, MRI or bone scintigraphy. Nuclear medicine methods include radioactive marked tracers such as Choline-PET imaging, each limited by a low specificity. PSMA-PET-imaging has been shown to be more sensitive as well as specific for PC staging [4–6].

Prostate-specific membrane antigen (PSMA) is a cell surface protein with high expression in PC cells [7]. At lower levels it is expressed within various organs such as salivary gland tissues and kidneys, and less so in liver, spleen, bowel and healthy prostate structures [8–10]. On the surface of PC cells it has been shown to be a thousand-fold increased compared to the other mentioned tissues [7]. In 2012, ^68^Ga-PSMA was developed as a novel PSMA-ligand and very recent studies show promising results for its usefulness in recurrent prostate cancer or as a staging tool [5, 9, 11]. Its introduction in diagnosis as well as treatment of PC is one of the most interesting developments in radiation oncology of urologic tumors.

However, the implementation of ^68^Ga-PSMA-PET-imaging in clinical routine is still only available in a few centers worldwide. Few data is available, especially on the value for staging and the impact on stage adaption during treatment planning for RT. The present work is to evaluate the integration of ^68^Ga-PSMA-PET-imaging into standard radiation planning of a primary definitive treatment of PC and to determine the influence on staging and on changes in the initially planned treatment concept for definitive RT in PC.

## Methods

Between August 2013 and April 2015, 15 patients were planned for definitive RT of the prostate with treatment planning based on CT, MRI and ^68^Ga-PSMA-PET-imaging at our institution. All patients gave written informed consent for the purpose of anonymized evaluation and publication of their data. All reported investigations were conducted in accordance with the Helsinki Declaration and with national regulations. The retrospective analysis was approved by the Ethics Committee of the Technical University Munich (permit 5665/13).

^68^Ga-PSMA-PET-imaging was regularly CT based. In one case (patient #3) MRI was selected because of better comparability with preceding images. Pre-treatment ^68^Ga-PSMA-PET CT or MRI was performed between June 2013 and October 2014 in the staging process. Patient characteristics are shown in Table 1. The procedure of elaboration and application of the ^68^Ga-PSMA-ligand complex has been described previously [12–14].

**Table 1:** Patients´ characteristics

**Median Age (years, range)**	74 (59–82)
**Gleason Score** ***n*** **(%)**	
Low risk (≤6)	5 (33.3)
Intermediate risk (7)	4 (26.7)
High risk (>7)	6 (40.0)
**Serum PSA (ng/ml)** ***n*** **(%)**	
Low risk (≤10)	7 (46.7)
Intermediate risk (10–20)	3 (20.0)
High risk (>20)	5 (33.3)
**Initial tumor stage (clinical examination and CT/MRI),** ***n*** **(%)**	
Biopsy T1c	7 (46.7)
Intermediate risk (T2b)	1 (6.7)
High risk (≥T2c)	7 (46.7)

Gleason score, PSA levels and Tumor stage risk group assignment according to the 2014 National Comprehensive Cancer Network guidelines on prostate cancer

All cases were discussed in an interdisciplinary panel of experienced radiation oncologists, radiologists, nuclear medicine physicians and urologists and treatment decisions were taken on consensus. Based on histo-pathological Gleason scores, pre-treatment PSA levels and clinical staging patients were assigned to risk groups (low, intermediate and high risk) according to the 2014 National Comprehensive Cancer Network guidelines on prostate cancer [15].

Retrospectively initial pre-^68^Ga-PSMA-PET tumor stages were classified according to the 2010 version AJCC/UICC staging system - including Gleason score, initial PSA levels, TNM stage, as well as a calculation of the Roach formula (risk of lymph node involvement [%] = 2/3 (PSA) + (GS-6) × 10) [16]. According to internal standard operation procedures of our department and following international guidelines, initial treatment decisions were made without the information obtained by ^68^Ga-PSMA-PET imaging based on CT/MRI as well as histo-pathological information available including PSA-level [3, 17]. After PSMA-imaging, all information was reviewed and re-classification was performed with the additional information taken into account. We evaluated the number of cases in which the information obtained by ^68^Ga-PSMA-PET imaging led to a change in staging and subsequently resulted in a change of the RT concept, such as additional irradiation of the lymph node regions or local dose escalations.

For treatment planning, CT scans with 3 mm slice thickness at full bladder and empty rectum were performed. For all patients presenting tumor stages cT1-cT3a the CTV definition included the prostate and the base of the seminal vesicles. For patients in stadium cT3b the seminal vesicles were included completely in the CTV. To obtain the PTV of the prostate 7 mm were added to the CTV in all directions. Patients with actual lymph node involvement (cN+) or an increased risk of an involvement (risk more than 20 % according to the Roach formula) received an irradiation of the pelvic lymph nodes. We defined the corresponding PTV of the lymph nodes, including the obturatory, internal and external iliac, common iliac and presacral (down to S3) lymph nodes, with a 5 mm margin [18]. If patients showed an increased uptake in ^68^Ga-PSMA-PET in defined lymph nodes, a simultaneously integrated boost was performed to the enhancement. The PTV of the suspected lymph node included the morphological correlate of the enhanced lymph node increased by at least 5 mm.

All patients received an intensity-modulated radiotherapy (IMRT): eight patients were treated with Tomotherapy® (Accuray, USA) and seven patients received an IMRT in RapidArc® technique by Varian, USA. The treatment was performed with full bladder and empty rectum under daily image guidance (IGRT).

## Results

A total of 15 patients were included into the present analyses. Data on imaging and on treatment decisions was followed prospectively through collaboration between the Departments of Nuclear Medicine, Urology and Radiation Oncology. Table 2 illustrates all tumor stages of enrolled patients; initial as well as after ^68^Ga-PSMA imaging. In 8 out of 15 patients (53.3 %) of all analyzed patients a change occurred in the TNM stadium due to the performed ^68^Ga-PSMA-PET examination. In 13 out of 15 patients (86.7 %) anti-hormonal treatment was given either before RT (80 %) or concurrently (6.7 %).

**Table 2:** Detailed information on each patient

Patient	Gleason score	PSA (ng/ml)	Roach formula risk (%)	Primary staging (CT = 1, MRI = 2, Choline-PET = 3)	cTNM without PSMA-PET			cTNM including PSMA-PET		
					T	N	M	T	N	M
1	6	19.1	13	1, 2, 3	1c	**1**	0	1c	**0**	0
2	7	8.5	16	1,2	**3a**	0	0	**3b**	0	0
3	8	9.6	26	1,2	2c	**X**	0	2c	**1**	0
4	6	39.6	26	1,2	3b	**0**	0	3b	**1**	0
5	9	34.2	53	1	1c	0	0	2b	0	0
6	6	8.2	6	1,2	2c	0	0	2c	0	0
7	7	36	34	1,2	3a	**0**	**0**	3a	**1**	**1** ^a^
8	6	10.8	7	1	1c	0	0	2a	0	0
9	7	23	25	1,2	3b	0	0	3b	0	0
10	8	9.4	26	1,2,3	2c	**1**	0	2c	**0**	0
11	8	13.5	29	1	**2b**	0	0	**3b**	0	0
12	7	8.7	16	1,2	1c	0	0	2a	0	0
13	6	63.9	43	1	1c	0	0	2c	0	0
14	9	9.6	36	1,2	1c	0	0	2c	0	0
15	8	23	35	1, 2	1c	**X**	X	2c	**1**	X^b^

Shown are biopsy Gleason score, maximum PSA level before treatment and calculation of lymph node involvement following the Roach formula, as well as evolvement of tumor stages before and after PSMA-PET imaging (changes are in bold). ^a^M1 based on para-aortal lymph nodes.^ b^PSMA uptake in the 5th rib right, without CT morphologic correlation, suggesting possible bone metastasis

### Down-staging

**Fig. 1:**
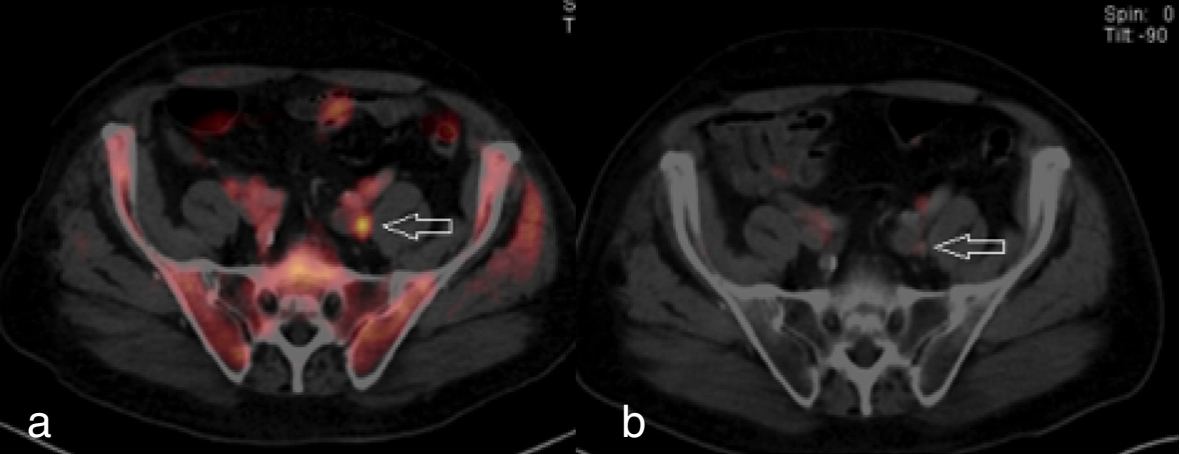
Down-staging by ^68^Ga-PSMA-PET. Choline-PET-CT (**a**) with elevated tracer uptake of left iliac lymph node. ^68^Ga-PSMA-PET (**b**) does not confirm the questionable involvement seen by Choline-PET-CT

Down-staging occurred in 2 cases (13.3 %). Those two cases were the only ones to receive a previous ^11^C-Choline-PET-CT. Radiologically positive lymph nodes were described on ^11^C-Choline-PET-CT, but did not show enhancement in the ^68^Ga-PSMA-PET scan and were therefore judged as not suspicious. In one patient (#1, Fig. 1a, b) multiple paraaortal and iliacal lymph nodes showed a strong uptake and morphological correlates in the previous Choline-PET-CT examination. In the ^68^Ga-PSMA-PET-CT, which was performed for clarification purposes, none of the nodes showed tracer accumulation. Thus, it was assumed that the disease was locally limited and the RT volume delineation included only the prostate. In another patient (#10), one particularly suspicious preacetabular located lymph node was identified among a few others that showed enhancement in a Choline-PET-CT. In the following ^68^Ga-PSMA-PET-CT imaging, no evidence at all was seen for lymph node involvement.

### Up-staging

**Fig. 2:**
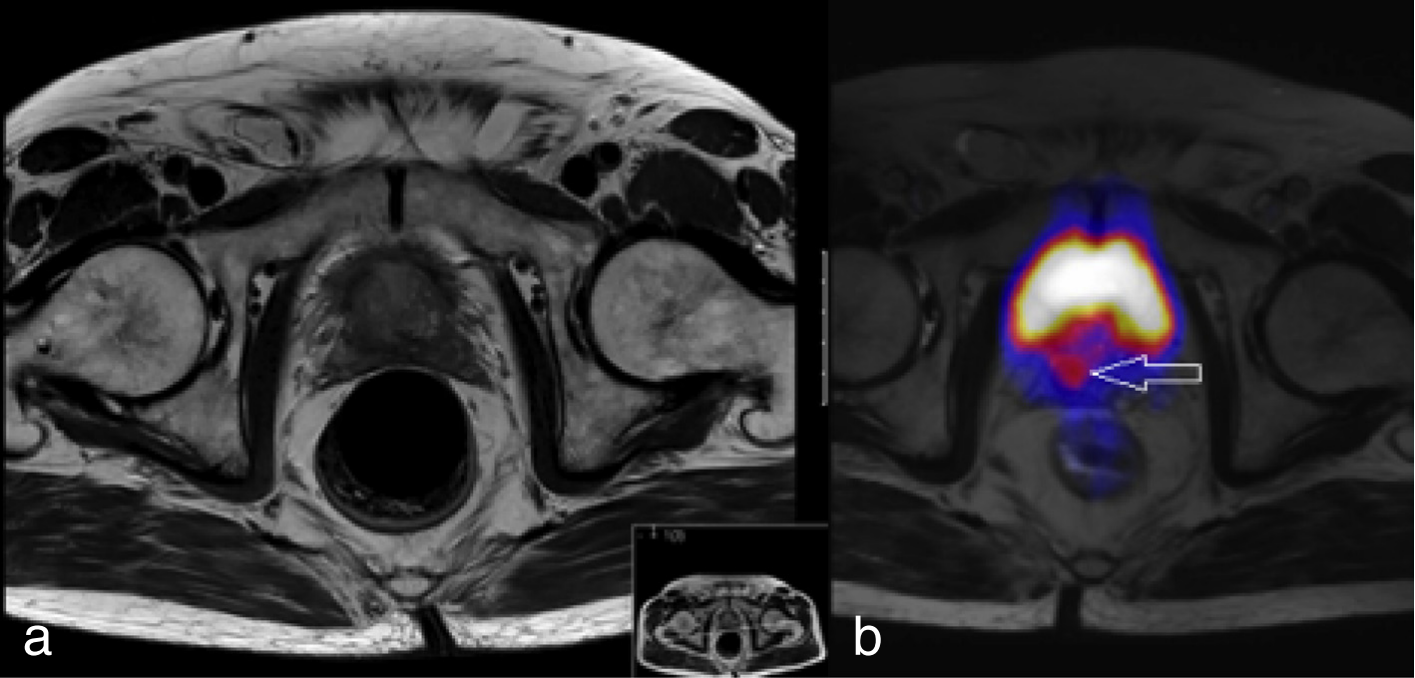
Up-staging due to extra capsular spread by ^68^Ga-PSMA-PET. Questionable extra capsular spread on MRI (**a**). In ^68^Ga-PSMA-PET imaging (**b**) is strong tracer uptake in the bladder and dorsal, as indicated by the arrow, in the extra capsular region, suggesting a strong possibility of its involvement

Up-staging was seen in 6 patients (40 %). In 2 cases the T stadium had to be corrected, and in another 4 patients a strong tracer uptake in initially unsuspicious lymph nodes was seen. To illustrate this, we shall highlight two cases. In the first case, Patient #2 had a primary tumor which was detected by MRI, but also showed a questionable extra capsular spread. The radiology colleagues described it as rather atypical for the tumor. Later a ^68^Ga-PSMA-PET-CT examination confirmed the extra capsular tumor spread on the dorsal right side with additional infiltration of the seminal vesicles. In this case the T stage evolved from cT3a to a cT3b (Fig. 2a, b).

**Fig. 3:**
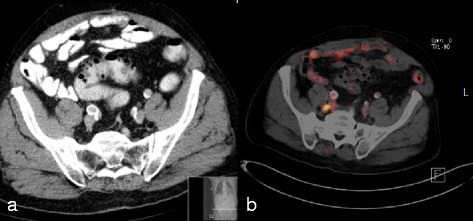
Up-staging due to lymph node involvement by ^68^Ga-PSMA-PET. Non-suspicious lymph nodes on MRI (**a**) presacral. In ^68^Ga-PSMA-PET imaging (**b**), the lymph node displays enhancement and was thus treated with a higher radiation dose

**Fig. 4:**
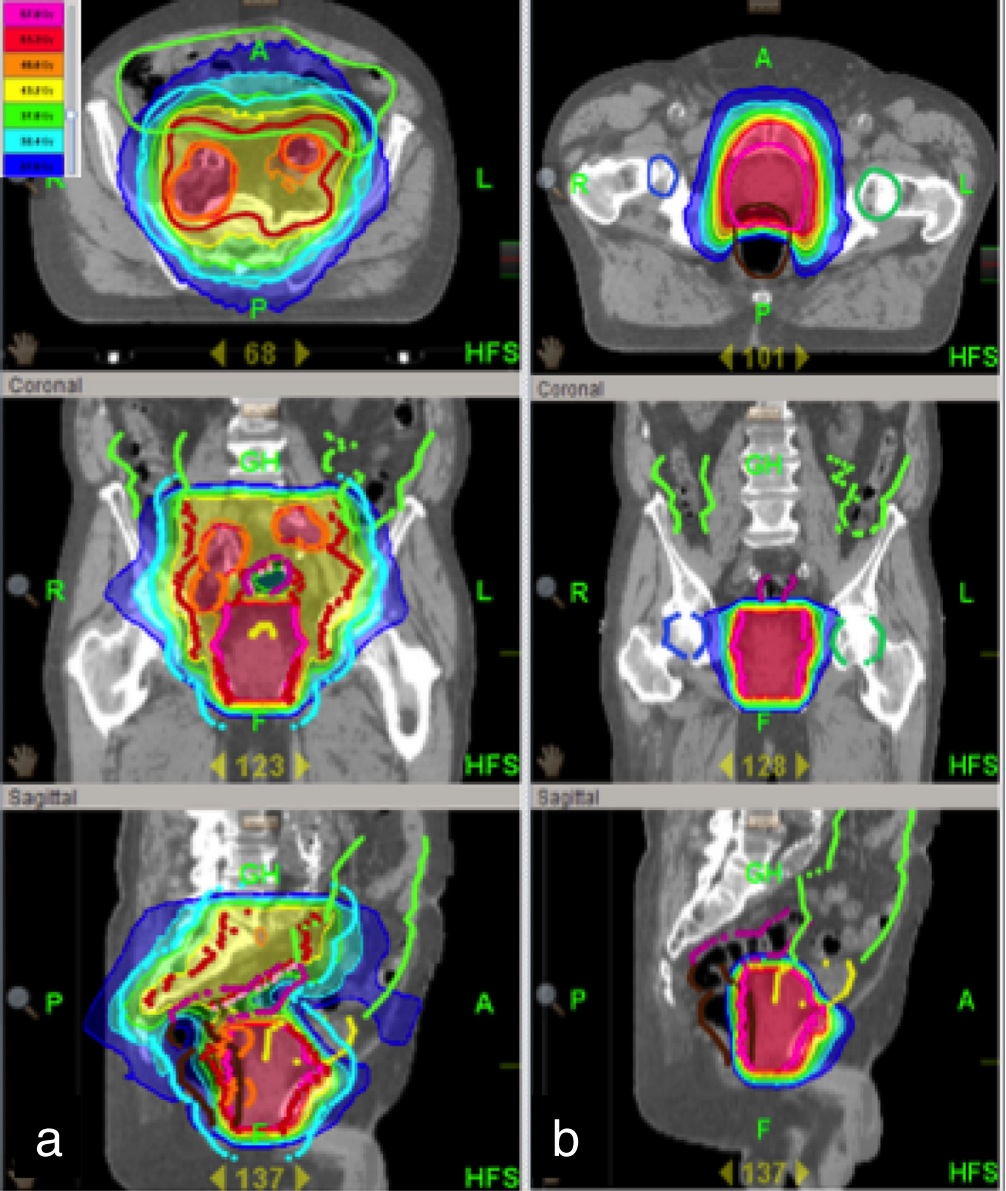
Treatment plan comparison. Basic treatment plan (**a**), including lymph node irradiation up to 45 Gy à 1.8 Gy. Based on the information obtained by ^68^Ga-PSMA-PET imaging, PET-positive nodes receive a simultaneous integrated boost to 54 Gy à 2.17 Gy. In comparison with Fig. 4a an example of the dose distribution (**b**) without the knowledge through the ^68^Ga-PSMA-PET; the lymph node involvement would not have been suspected and therefore only the prostate itself would have been treated

In the second case, neither MRI nor CT found any indication of lymph node metastases in patient #4, but after ^68^Ga-PSMA-PET-CT examination of one pararectal left lymph node, another presacral right (Fig. 3a, b), and several others along the iliacal vessels, a pronounced tracer uptake was shown. Without the positive lymph nodes seen in the PET examination, the pelvic lymphatic drainage would have been irradiated due to the risk of lymph node involvement of 26 % according to the Roach formula. Including the newly obtained information the affected lymph nodes were treated with a higher radiation dose (54 à 2.17 Gy as a simultaneously integrated boost) than the surrounding pelvic lymphatics (45 à 1.8 Gy). The treatment plan is illustrated in Fig. 4a. The primary tumor within the prostate received no higher dose and the whole prostate plus seminal vesicles were treated to a total dose of 74 Gy.

### No change of stage

Seven patients (46.7 %) experienced no change of stage. This also included those cases where the biopsy confirmation (T1c) evolved to any other T-stage based on image modalities.

As shown in Table 3, advanced tumors with initially higher T-stages have an increased risk of upstaging by ^68^Ga-PSMA imaging. On the other hand small tumors (lower T-stages) were more likely to experience down-staging through ^68^Ga-PSMA-PET.

**Table 3:** Distribution of prostate cancer T stage before and after PSMA-PET imaging of the prostate

T stage	Initial clinical Staging: *n* (%)	Relative up-staging by PSMA-PET-imaging: *n* (%)	Relative down-staging by PSMA-PET-imaging: *n* (%)	PSMA-PET-imaging staging: *n* (%)
Biopsy T1c	6 (40.0)	1 (6.7)	1 (6.7)	0
Low risk (≤T2a)	0	0	0	2 (13.3)
Intermediate risk (T2b)	2 (13.3)	1 (6.7)	0	1 (6.7)
High risk (≥T2c)	7 (46.7)	4 (26.7)	1 (6.7)	12 (80.0)

Similarly, the Gleason score seemed to influence the likelihood of a tumor up-staging. With a Gleason score of 7 or under, only 1 patient (6.7 %) experienced a further up-staging. For scores of 7 or over, 2 (13.3 %) and respectively 3 (20 %) cases had to be grouped into a higher TNM stadium. Down-staging worked up for Gleason score was balanced for patients with scores over and lower than 7. Concerning PSA levels the trend seemed to be less convincing. The six up-stagings were spread out over three groups. 3 cases were found for PSA levels under 10 ng/ml and one case for PSA levels between 10 and 20 ng/ml. 2 cases occurred in patients having initial PSA levels over 20 ng/ml. Nevertheless, down-staging was seen in 2 patients with PSA levels under 20 ng/ml, as seen in Table 4.

**Table 4:** Dependence on Gleason Score and PSA serum levels for up- and down-staging

	Number	Tumor up-staging: *n* (%)	Tumor down-staging: *n* (%)
Gleason score <7	5	1 (6.7)	1 (6.7)
Gleason score 7	4	2 (13.3)	0
Gleason score > 7	6	3 (20.0)	1 (6.7)
PSA level <10 ng/ml	7	3 (20.0)	1 (6.7)
PSA level 10–20 ng/ml	3	1 (6.7)	1 (6.7)
PSA level >20 ng/ml	5	2 (13.3)	0

The radiotherapy concept was changed in 33.3 % of the patients analyzed. This produced relevant changes in the gross tumor volume and clinical target volume. Among these changes an additional irradiation of the pelvic lymph drainage due to tracer uptake in lymph nodes was performed in 25 %. Furthermore, boost volumes on positive lymph nodes detected by ^68^Ga-PSMA-PET were added in 80 % of these cases (Fig. 4a and b).

## Discussion

^68^Ga-PSMA-PET has shown high diagnostic accuracy for patients with PC. The data from the present manuscript show that ^68^Ga-PSMA-PET imaging had a huge impact on staging. Thus, compared to standard imaging based on pathological parameters, PSA values and imaging with CT and/or MRI only, a substantial impact on tumor staging and re-staging can be expected based on ^68^Ga-PSMA-PET imaging. Since in definitive RT for PC precise delineation of involved tissue as well as tissue at risk is necessary, and since there is a clear benefit of dose escalations to involved tissue, the diagnostic value of ^68^Ga-PSMA PET is evident.

Recently, the usefulness and accuracy of ^68^Ga-PSMA-PET imaging has been described increasingly in the recurrent setting of PC [5, 11, 19, 20]. For example, Eiber et al. [5] attested significantly higher detection rates of PC and lymph nodes as previously reported for other imaging modalities. This particularly applied in the range of low PSA-levels (<0.5 ng/mL). In 33 % of cases, the tumor site was exclusively detected by ^68^Ga-PSMA-PET examination. An additional 25 % of the patients showed lesions that were not detectable by CT. In total, over 50 % of crucial information was identified by ^68^Ga-PSMA-PET for final diagnosis, as well as staging and treatment decisions. The specificity of ^68^Ga-PSMA-PET has been demonstrated by the use of PSMA-radioguided surgery as well as for lymph node staging in primary PC [21, 22]. With the radiotherapy concept changing in a third of all patients we assume that a better sensitivity and specificity may lead to improved radiotherapy concepts in patients having received a ^68^Ga-PSMA-PET.

All ^68^Ga-PSMA PET examinations were performed for treatment planning on the premises of ^68^Ga-PSMA-PET imaging as a “gold standard”. For precise diagnosis, correlation with pathological analyzes is necessary. This is currently being done for the diagnostic value of lymph node identification within a prospective trial at our institution. Though more data is still needed to verify sensitivity and specificity of the ^68^Ga-PSMA-PET, promising studies have been conducted. A current study by Maurer et al. [23] showed for primary patients before radical prostatectomy and patients who underwent surgery for lymph node metastases a high histo-pathologically proven sensitivity (75 and 65.9 %) and specificity (98.8 and 98.9 %). CT or MRI imaging sensitivity (41.7 and 43.9 %) and specificity (85.5 and 85.4 %) was clearly lower. Another recent study by Giesel et al. confirms these findings [24].

It should also be mentioned that PSMA-negative PC seems to be rare, but false negative cases have been reported in the literature [8, 20, 25]. Whether this approach is correct will be verified by long-term PSA-levels. At our institution PSA-levels are monitored every 3 months after radiation therapy.

Nevertheless, in previous studies other imaging modalities such as CT and MRI showed similar performances in PC nodal staging by indirectly assessing nodal invasion measuring lymph node diameter. Consequently, their sensitivity was proven to be low. Using a 10 mm threshold, the sensitivity was reported to be less than 40 % [26]. Functional PET imaging, mostly using radiolabeled-choline derivatives, are of limited value and often underestimate the extent of metastatic spread [27]. Evangelista et al. [28] evaluated, in a big meta-analysis, a high specificity for ^11^C-Choline-PET CT of 95 % in primary lymph node staging but a low sensitivity of just 50 %. Husarik et al. [29] found a sensitivity to detect recurrent disease of 86 % and described these results as rather discouraging, especially in terms of its inability to detect small metastases - recurrent disease was reliably diagnosed in patients with PSA levels of >2 ng/ml. In another study Choline-PET-CT was found to be statistically significantly inferior when compared to ^68^Ga-PSMA-PET-CT in a recurrent setting as described by Afshar-Oromieh et al. [24].

Interestingly, there have been studies that support the theory that detection rates increase parallel with elevated PSA levels [30]. In a primary setting a tailored treatment approach is very important, and with mostly considerably elevated PSA levels, ^68^Ga-PSMA-PET imaging could prove to be overly effective. Here, the measurement of PSA level alone can at best calculate the risk of lymph node metastases by using the Roach formula [16]. On a similar note, it has been noted that there is higher expression in lesions with higher Gleason scores [5, 31]. The Gleason score as well as PSA levels were also taken into account in our study.

One main limitation of this analysis is its small patient number. However, since no data are currently available and ^68^Ga-PSMA-PET imaging is performed at several centers, the results provide a useful basis for decision-making in radiation oncology. Additionally, the homogenous results in the present “typical“ PC patient population underline the reliability of the reported data. It is important to note that in patients with higher T-stages, as well as higher PSA-values, the impact of ^68^Ga-PSMA-PET is larger on restating than in other tumor stages, meaning that in those patients a larger amount of up-stagings were observed than for lower tumor stages.

However, other factors such as anti-hormonal treatment might influence staging changes and therefore represent limitations to our study. In both of the down-staging cases anti-hormonal treatment was given either at the time or before ^68^Ga-PSMA imaging. These factors possibly had an effect on PSMA-image enhancement and subsequent down-staging. In contrast, Afshar-Oromieh et al. [11] showed that patients taking anti-hormonal medication at the time of the ^68^Ga-PSMA-PET examination had more often positive PET results than patients not receiving hormonal therapy. Also, in some cases the time span between initial imaging modalities and ^68^Ga-PSMA-PET was up to a few months (maximum of 3 months - e.g. due to bridging anti-hormonal treatment). Therefore the possibility cannot be excluded that tumor stages in a few cases not only evolved because of the diagnostic tool, but also simply because of time.

In summary, we see great potential for ^68^Ga-PSMA-PET imaging. On one hand, in the case of down-staging, patients are potentially spared from unnecessary toxicities to surrounding tissues due to smaller radiation fields. For example, radiation doses could be drastically reduced by only treating lymphatic drainage if pelvic enhancements are obtained by ^68^Ga-PSMA-PET and not based on the calculated lymph node risk according to the Roach formula. In order to standardize this procedure at least PSA-long-term observations have to be conducted. On the other hand, in the case of up-staging, enlarged radiation volumes were its consequence and additional radiation dose (boost) to affected lymph nodes or within the prostate region could potentially translate into improved local control and/or overall survival for our patients. This will be evaluated within a prospective clinical trial at our institution.

## Conclusions

The integration of ^68^Ga-PSMA-PET imaging into RT treatment planning can be a powerful tool and useful method for detailed target volume delineation. In addition to the indication for PSA persistence after radical prostatectomy or PSA relapse without image morphological correlate, a ^68^Ga-PSMA-PET can also be advantageous for radiation treatment planning in primary PC. Assuming ^68^Ga-PSMA-PET as “gold standard” we showed in this initial patient series that the implementation of ^68^Ga-PSMA -PET hybrid imaging frequently led to changes in the TNM staging and consequently influences the radiotherapeutic treatment regimen as well as the clinical target volumes. This yields the possibility for boost volumes directed to PET-positive areas. Whether this will be reflected by an improvement in survival rates needs to be investigated in larger prospective studies.
